# Trends and determinants of adolescent pregnancy: Results from Kenya demographic health surveys 2003–2014

**DOI:** 10.1186/s12905-022-01986-6

**Published:** 2022-10-10

**Authors:** Lilian Mutea, Vincent Were, Susan Ontiri, Kristien Michielsen, Peter Gichangi

**Affiliations:** 1 Department of Public Health and Primary Care, Faculty of Medicine and Health Sciences, Ghent University, Ghent, Belgium; 2 2U.S. Agency for International Development (USAID) Kenya and East Africa, Washington, DC USA; 3 Kenya Medical Research Institute (KEMRI),, Nairobi,, Kenya; 4 Population Services International, Washington, DC USA; 5 Technical University of Mombasa, Mombasa, Kenya

**Keywords:** Trends, Determinants, Adolescent, Pregnancy, Demographic, Health Survey, Reproductive health

## Abstract

**Background:**

Adolescent pregnancy increases the risk of disability and death due to unsafe abortion, prolonged labour and delivery, and complications after birth. Availability of accurate data is important to guide decision-making related to adolescent sexual reproductive health (ASRH). This study analyses the trends in prevalence and factors associated with adolescent pregnancy in Kenya using data from three national Demographic Health Surveys (2003, 2008/2009, 2014).

**Methods:**

Our analysis focused on a subsample of data collected from women aged 20 to 24 years. A trend analysis was performed to establish a change in the rate of adolescent pregnancy in 2003, 2008/2009, and 2014 survey data points. Binary Logistic regression and pooled regression analysis were used to explore factors associated with adolescent pregnancy.

**Results:**

The percentage of women aged 20 to 24 years who reported their first pregnancy between ages 15 and 19 years was 42% in 2003 and 42.2% in 2009 but declined to 38.9% in 2014. Using regression analyses, we established that education status, marital status, religion and wealth quintile were associated with adolescent pregnancy. Trend analysis shows that there was an overall decreasing trend in adolescent pregnancy between 2003 and 2014.

**Conclusion:**

Although Kenya has made strides in reducing the prevalence of adolescent pregnancy in the last decade, much more needs to be done to further reduce the burden, which remains high.

**Definition:**

Adolescents: Although WHO defines the adolescence period as being 10–19 years, this paper focuses on the late adolescent period, 15–19 years, here in referred to as adolescents.

**Supplementary Information:**

The online version contains supplementary material available at 10.1186/s12905-022-01986-6.

## Background

Globally, complications during pregnancy and childbirth are the leading cause of death among women aged 15 to 19 years [1]. Adolescent pregnancy increases the risk of complications of unsafe abortion, prolonged labour, delivery, and the postnatal period [2, 3]. Babies born to adolescent mothers face higher risks of low birth weight, preterm delivery, and severe neonatal conditions [4].

Adolescent pregnancy is more likely to occur in marginalized communities, commonly associated with low socioeconomic status, lack of education, and limited employment opportunities [5]. Adolescent pregnancy is also strongly associated with poor access to health services, non-use of contraception, and early sexual initiation [5]. The early adolescent sexual activity contributes to unintended adolescent pregnancy and increases young people’s risk of infection with HIV and other sexually transmitted diseases (STIs) [6]. Sexual activity among adolescents is highest across sub-Saharan Africa compared with other regions, a reflection which is linked to the higher rates of child marriage in the region [7]. Findings from the Performance Monitoring and Accountability Framework (PMA 2020) show that in 2018, 48.2% of women aged 18–24 years had their first sexual encounter before 18 years [8]. Some possible explanation is that child marriage is a common practice in many regions of Kenya which contributes to the high adolescent pregnancy rates.[9].

Although very early childbearing (giving birth before age 15 years) declined globally between 2006 and 2015, childbearing among older adolescents (between 15 and 19 years) has remained stagnant, especially in sub-Saharan Africa [10].

Based on data from the late 1990s from Burkina Faso, Cameroon, Côte d’Ivoire, Guinea, and Togo, pregnancy among girls in school contributes to school dropout and/or child marriage [11]. Using data from the late 1990s, Lloyd and Mensch found that for girls aged 15 to 24, child marriage and early pregnancy directly account for between 5% and 33% of dropouts, depending on the country [12]. Based on their subsequent lower education attainment, many girls who marry early have fewer employment opportunities, often perpetuating a cycle of poverty. Increasing the age at first birth is therefore important as it both positively impacts the health status of the young mother and baby and improves the young girl’s future—socially, emotionally, and economically [13].

Kenya’s 2015 adolescent sexual and reproductive health (ASRH) policy provides an enabling legal and socio-cultural environment to ensure that adolescents have access to SRH information and services through strengthened intersectoral coordination, partnership and community participation, improved data collection and analysis, and generation of age- and sex-disaggregated data on adolescents [14]. From 2003 to 2008/2009, Kenya recorded a reduction in its adolescent pregnancy rate from 23 to 18%; it remained at 18% in 2014 [15–17]. These figures are likely an underestimation as they omit the number of pregnant adolescents who may not have carried the pregnancy to term.

Comprehensive data on trends, patterns, and prevalence of adolescent pregnancy are required to design evidence-based interventions and target them effectively. Country-specific estimates of pregnancy and birth among adolescents can also motivate policy and programmatic responses to teen pregnancies and help monitor progress toward reducing their incidence [18]. Even where incidence is low, evidence shows that data on adolescent pregnancies can highlight the unmet need for information and services to help adolescents prevent unintended pregnancies [18]. Kenya has a target to reduce the pregnancy rate among adolescent girls aged 15–19 years to 10% in 2025. Regular monitoring of progress is important to establish if the country is on track to achieve this target [14]. To meet this need, we undertook a secondary analysis of data from Kenya Demographic Health Surveys (KDHS) conducted at three points: 2003, 2008/2009, and 2014 using data from young women aged 20–24 years. The data points align with key policy milestones for ASRH programming in Kenya that began with development of the first ASRH policy in 2003. The policy marked the beginning point for ASRH programs and recommendations from this study would be relevant for future ASRH programming in Kenya. This study aims to establish trends in adolescent pregnancy, as well as the factors associated with pregnancy among adolescents aged 15–19 years old.

## Methodology

### Study design

The KDHS is a national cross-sectional survey whose objective is to provide the country with reliable information and analyses useful for guiding informed policy choices. Through analysis of KDHS data from three surveys (2003, 2008/2009, and 2014), this study identifies trends in the prevalence of adolescent pregnancy as well as factors associated with adolescent pregnancy in Kenya. In doing so, it demonstrates how DHS data can be used to generate additional information using quantitative analysis methods not applied in the DHS final reports.

### Study variables

The outcome of the study was pregnancies which occurred between ages 15 and 19. The independent variables included in the analysis were the social demographic factors collected in the DHS, which are also determinants of adolescent pregnancy identified in other similar studies [19, 20] (REF). which include highest education level attained (no education, incomplete primary level, primary, secondary, and higher) residence (urban and rural), religion (Catholic, Protestant, Muslim, others, and none), marital status (single, married, previously married), and household wealth which was calculated using the DHS five wealth quintile that uses the household asset data collected in the Household Questionnaire. The quintiles are then generated through a principal components analysis.

### Study population

In each DHS, the subpopulation of women aged 15–19 is composed of respondents who may not have experienced a pregnancy at the time of their interview but could still experience this outcome as adolescents at a later date since they were still within the adolescent years. Therefore, the analysis focused on the subpopulation of women who most recently completed adolescence (i.e., women aged 20–24), since all women in this subpopulation were past the age at which adolescent pregnancy was possible. Although WHO defines the adolescence period as being 10–19 years, this paper focuses on the late adolescent period, 15–19 years, here in referred to as adolescents. Data on pregnancy levels during the early adolescent stage, 10–14 years was omitted due to the extended recall period and to be consistent with other similar studies that have used this approach of restricting analysis to women to pregnancy levels of 15–19 years [19, 20]. However, we have presented early pregnancy data as a supplemental file number one. This analysis, focusing on prior experiences of women during adolescence and focus on older adolescents are both limitations of this study.

### Sample size

DHS data were collected from 8561 household in 2003 (96% response rate), 8195 females aged 15–49 (94% response rate); in 2008/9, 9057 households(98% response rate), 8444 females (96% response rate), and in 2014, 17,409 households were included (99% response rate), 14,741 females (96% response rate).This study uses data collected from the women’s questionnaire in the DHS and analysis was limited to women aged 20–24 years (2003: n = 1,691; 2008/2009: n = 1,715; and 2014: n = 5,735).

### Data source

The KDHS data sets used in this study are available at https://dhsprogram.com/data/available-datasets.cfm. KDHS data are collected through the administration of structured questionnaires by trained enumerators at the household level. Data entry is done using the Census and Survey Processing System (CSPro) software (https://www.census.gov/data/software/cspro.html). Through a collaboration of the Kenya Bureau of Statistics (KNBS) and the DHS Program, the data are cleaned, coded, and made publicly available.

### Data analysis

The DHS data was stratified, a process by which the sampling frame was divided into subgroups or strata that are as homogeneous as possible using certain criteria. The weighted analysis was conducted in the three DHS surveys to correct for over-sampling in certain strata and other non-sampling error. The weights were obtained by dividing the woman’s sample weight by 1,000,000. The sampling and weighting techniques were similar for all the three years. The analysis accounted for stratification, clustering, and weighting. The three KDHS data sets were analysed using Stata version 14 [21]. The secondary analysis covered the following aspects:

- **Descriptive statistics**: Adolescent pregnancies were tabulated against sociodemographic characteristics for the survey years 2003, 2008/2009, and 2014. To achieve this, the variables, education, residency, marital status, religion, and household wealth quintile were tabulated to obtain the frequency and percentage of women in each category. Wealth quintile was already pre-assigned in the DHS data.
- **Trends analysis**: A multivariable logistic regression was used to check for trend across the three survey years. An odds ratio of less than 1 indicated decreasing trend while an odds ratio of greater than 1 indicated an increasing trend. 95% Confidence interval was also reported. Adolescent pregnancy was coded 1 if the respondent reported pregnancy and 0 if the respondent did not. Age of pregnancy was cross tabulated against sociodemographic characteristics and prevalence was obtained and reported at a 95% confidence interval (CI).
- **Multivariate regression analysis**: The outcome of interest of our regression analysis was a pregnancy in adolescents, which was coded 1 if the respondent reported a pregnancy between ages 15 and 19 years and 0 if they had not. Bivariate and multivariate regression analysis was performed to establish factors associated with adolescent pregnancy. The study used backward regression, whereby the outcome variable was regressed against the independent variables. Factor variables whose odds ratio did not overlap 1.0 were considered to be significant.
- **Pooled regression analysis**: For pooled analysis, data from 2003, 2008/2009, and 2014 were appended to form one data set (N = 9,141) and the survey year was treated as an independent variable. The outcome variable remained pregnant during adolescence years. Bivariate and multivariate regression was applied as described previously.

The variance inflation factor (VIF) was used to test for multicollinearity between education and wealth quintile. The mean VIF was 1.28 in 2003, 1.36 in 2008, and 1.35 in 2014. We conclude that there is no multicollinearity since the mean VIF across the three surveys is below the recommended threshold of 10 [22].

### Ethical approval

The authors obtained permission from the DHS Program to access the data sets. DHS program already obtains approval at the point of data collection for each DHS. For this secondary analysis, the study was guided by a protocol approved by the Kenya Medical Research Institute research ethics review committee number 597.

## Results

A total of 9151 women aged 20–24 years were included. Table 1 describes the sociodemographic characteristics of women aged 20–24 in 2003, 2008/2009, and 2014 KDHSs. As the Table 1 shows, the majority were from rural areas, had attained secondary or higher education, were married/living with their partner, were from the highest wealth quintile, and were Protestant/other Christian.

**Table 1:** Sociodemographic characteristics of women aged 20–24 years

	2003		2008/2009		2014	
**Sociodemographic**	**n**	**%**	**N**	**%**	**n**	**%**
**Residence**						
Urban	524	31	539	31.4	2,769	48.3
Rural	1,168	69	1,176	68.6	2,966	51.7
**Education**						
No education	124	7.3	124	7.2	301	5.2
Primary incomplete	468	27.7	426	24.9	1,015	17.7
Primary complete	521	30.8	504	29.4	1,353	23.6
Secondary+	578	34.2	661	38.5	3,066	53.5
**Marital status**						
Never married	612	36.2	651	37.9	2,225	38.8
Married/living with partner	965	57	958	55.8	3,133	54.6
Widowed/divorced/separated	114	6.8	106	6.2	377	6.6
**Household wealth quintile**						
Lowest	220	13	253	14.7	809	14.1
Second	264	15.6	267	15.6	973	17
Middle	294	17.4	296	17.3	998	17.4
Fourth	363	21.5	353	20.6	1,290	22.5
Highest	550	32.5	546	31.8	1,665	29
**Religion**						
Catholic	411	24.3	359	20.9	1,187	20.7
Protestant	1,125	66.5	1,190	69.5	4,091	71.3
Muslim	115	6.8	122	7.1	351	6.1
No religion	33	1.9	38	2.2	89	1.6
Other (e.g., traditional)	7	0.4	3.4	0.3	17	0.3
**Total**	1,691	100	1,715	100	5,735	100

### Adolescent pregnancy among women aged 20–24 years

Table 2 shows the percentage of women aged 20–24 years who were pregnant, got pregnant or experienced pregnancy during the adolescent period by age. Overall, there was a decreasing trend in adolescent pregnancy by age between 2003 and 2014(AOR = 0.84, 95% CI = 0.73–0.98). Between 2003 and 2014, there was an increase of 1.4% points in the pregnancy rate among 15-year-olds. A decrease of 0.1%, 1.3% and 3.1 was noted in the percentage of women reporting adolescent pregnancy at ages 17, 18, and 19 years from 2003 to 2014.

**Table 2:** Percentage of women aged 20–24 years by age

	2003 (N = 1,691)		2008/2009 (N = 1,715)		2014 (N = 5,735)		
**Age**	**n**	**% (95% CI)**	**n**	**% (95% CI)**	**n**	**% (95% CI)**	**Trend 2003–2014**
15	37	2.2(1.6-3.0)	66	3.8(3.0-4.9)	206	3.6(3.1–4.1)	0.84(0.73–0.98)
16	164	9.7(8.4–11.2)	181	10.5(9.2–12.1)	589	10.3(9.5–11.1)	
17	326	19.3(17.5–21.3)	366	21.4(19.5–23.4)	1,102	19.2(18.2–20.2)	
18	507	30.0(27.8–32.2)	564	32.9(30.7–35.2)	1,645	28.7(27.529.9)	
19	710	42.0(39.6–44.3)	724	42.2(39.9–44.6)	2231	38.9(37.6–40.2)	

**Table 3:** Percentage of women aged 20–24 years who experienced pregnancy during adolescence (ages 15–19 years)

	2003 (N = 1,691)	2008/2009 (N = 1,715)	2014 (N = 5,735)						
	**N**	**% (95% CI)**	**n**	**% (95% CI)**	**n**	**% (95% CI)**		**Trend 2003–2014**	
Prevalence of pregnancy (15–19)	710	42.0(39.6–44.3)	724	42.2(39.9–44.6)	2231	38.9(37.6–40.2)		0.88(0.76–1.02)	
**Residence**									
Urban	163	31.2(27.4–35.3)	154	28.7(25.0-32.6)	867	31.3(29.6–33.1)			
Rural	547	46.8(43.9–49.7)	570	48.5(45.6–51.3)	1364	46.0(44.2–47.8)			
**Education**									
No education	65	52.5(43.7–61.2)	78	63.0(54.2–71.1)	165	54.7(49.1–60.3)			
Primary incomplete	303	64.7(60.2–68.9)	273	64.0(59.3–68.4)	678	66.8(63.9–69.6)			
Primary complete	216	41.5(37.4–45.8)	240	47.7(43.4–52.1)	694	51.3(48.6–53.9)			
Secondary+	126	21.7(18.5–25.2)	133	20.2(17.3–23.4)	694	22.6(21.2–24.1)			
**Household wealth quintile**									
Lowest	141	63.7(57.1–69.8)	151	59.5(53.3–65.4)	467	57.8(54.3–61.1)			
Second	140	53.2(47.1–59.2)	143	53.6(47.6–59.5)	**506**	52.0(48.8–55.1)			
Middle	138	46.9(41.2–52.6)	139	47.1(41.5–52.8)	440	44.1(41.0-47.2)			
Fourth	130	35.9(31.1–41.0)	140	39.6(34.6–44.8)	431	33.4(30.9–36.0)			
Highest	161	29.2(25.6–33.2)	151	27.7(24.1–31.6)	387	23.2(21.3–25.3)			
**Religion**									
Catholic	162	39.5(34.8–44.3)	152	42.3(37.3–47.5)	411	34.6(31.9–37.4)			
Protestant/Other Christian	465	41.3(38.5–44.2)	488	41.0(38.2–43.8)	1615	39.5(38.0–41.0)			
Muslim	58	50.6(41.5–59.6)	59	48.2(39.4–57.0)	138	39.5(34.5–44.7)			
No religion	22	66.0(48.2–80.2)	23	62.1(45.7–76.1)	60	67.0(56.5–76.0)			
Other	3	38.8(11.5–75.7)	2	38.5(8.6–80.6)	7	40.1(19.9–64.4)			
**Marital status**									
Never married	82	13.3(10.8–16.3)	97	15.0(12.5–18.0)	317	14.2(12.9–15.8)			
Married/living with partner	565	58.6(55.5–61.7)	555	57.9(54.8–61.0)	1670	53.9(52.2–55.7)			
Widowed/divorced/separated	63	54.7(45.5–63.6)	72	67.5(58.0-75.8)	224	59.4(54.3–64.2)			

Table 3 shows the overall trends in adolescent pregnancy rates based on the experiences of women aged 20–24 years who were interviewed during the three DHSs. Overall, there was a decreasing trend between 2003 and 2014 in adolescent pregnancy among women aged 20–24 years (AOR = 0.88, 95% CI = 0.76–1.02). Between 2003 and 2014, there was an increase of 9.8% points in women who had completed primary education and became pregnant during adolescence period 15–19 years. Adolescent pregnancy in the highest wealth quintile declined by 6% points between 2003 and 2014.

### Factors associated with adolescent pregnancy among women aged 20–24 years

Using multivariate logistic regression, we analysed the factors associated with adolescent pregnancy and compared them over time (Table 4). Results showed that education level and marital status and wealth quintile were associated with adolescent pregnancy in 2003 and 2014 while in 2008/9 only education and marital status was significant. In 2003, adolescent women who did not complete their primary education were almost twice as likely to become pregnant in the teenage years than those who never went to school (AOR = 1.92, 95% CI = 1.12–3.29). The odds were also much higher among married (AOR = 6.53, 95% CI = 4.85–8.80) and divorced/separated (AOR = 4.89, 95% CI = 2.87–8.33) adolescents than among the unmarried category. The odds for reporting pregnancy in adolescent years were highest among the lowest wealth quintile (AOR = 2.49, 95% CI = 1.46–4.22) followed by the second (AOR = 1.80, 95% CI = 1.13–2.88) and the middle quintiles (AOR = 1.61, 95%CI = 1.04–2.50) as compared to the highest wealth quintile.

In 2008/2009, women with a secondary school education were less likely to report pregnancy during adolescence (AOR = 0.28, 95% CI = 0.15–0.50). Once again, marital status was a factor; married (AOR = 5.33, 95% CI = 3.69–7.69) and divorced (AOR = 8.67, 95% CI = 4.30–17.48) women were more likely to report pregnancy during adolescence than unmarried women.

In 2014, women who had not completed primary education (AOR = 1.88, 95% CI = 1.33–2.66) had higher odds of reporting adolescent pregnancy, whereas those with secondary education (AOR = 0.50, 95% CI = 0.36–0.71) had lower odds. Compared to unmarried women, married (AOR = 5.14, 95% CI = 4.19–6.29) and divorced (AOR = 6.13, 95% CI = 4.26–8.83) women had higher odds. Women in the lowest (AOR = 1.97, 95% CI = 1.43–2.72), second (AOR = 1.86, 95% CI = 1.39–2.50), and middle (AOR = 1.90, 95% CI = 1.42–2.55) wealth quintiles all had higher odds of reporting pregnancy by age 19 years than women in the highest wealth quintile.

**Table 4:** Regression analysis of socio-demographic characteristics for women aged 20–24 years who experienced pregnancy during adolescence in 2003, 2008–2009, and 2014

	2003 (N = 1,691)		2008/2009 (N = 1,715)		2014 (N = 5,735)	
	**COR (95% CI)**	**AOR (95% CI)**	**COR (95% CI)**	**AOR (95% CI)**	**COR (95% CI)**	**AOR (95% CI)**
**Residence**						
Urban	0.52(0.39–0.68)	1.24(0.85–1.82)	0.43(0.28–0.65)	0.73(0.44–1.20)	0.54(0.46–0.63)	1.01(0.83–1.24)
Rural	Ref	Ref	Ref	Ref	Ref	Ref
**Education**						
No education	Ref	Ref	Ref	Ref	Ref	Ref
Primary incomplete	1.66(1.08–2.54)	1.92(1.12–3.29)	1.04(0.63–1.73)	0.98(0.58–1.66)	1.67(1.27–2.19)	1.88(1.33–2.66)
Primary complete	0.64(0.41–0.99)	1.08(0.61–1.92)	0.53(0.35–0.82)	0.59(0.34–1.04)	0.87(0.67–1.13)	1.19(0.84–1.68)
Secondary +	0.25(0.16–0.39)	0.61(0.34–1.11)	0.15(0.09–0.23)	0.28(0.15–0.50)	0.24(0.19–0.31)	0.50(0.36–0.71)
**Marital status**						
Never married	Ref	Ref	Ref	Ref	Ref	Ref
Married/living together	9.22(6.88–12.36)	6.53(4.85–8.80)	7.79(5.35–11.36)	5.33(3.69–7.69)	7.05(5.81–8.55)	5.14(4.19–6.29)
Div/Sep/Widowed	7.86(4.66–13.27)	4.89(2.87–8.33)	11.76(5.98–23.12)	8.67(4.30-17.48)	8.80(6.18–12.53)	6.13(4.26–8.83)
**Religion**						
Roman catholic	0.34(0.15–0.74)	0.52(0.22–1.26)	0.45(0.21–0.96)	1.27(0.39–4.13)	0.26(0.14–0.47)	0.84(0.47–1.49)
Protestant	0.36(0.17–0.77)	0.63(0.27–1.44)	0.42(0.20–0.90)	1.23(0.39–3.94)	0.32(0.18–0.57)	0.81(0.47–1.40)
Muslim	0.53(0.22–1.24)	0.52(0.20–1.32)	0.57(0.26–1.26)	0.81(0.27–2.38)	0.32(0.18–0.59)	0.61(0.34–1.10)
No religion	Ref	Ref	Ref	Ref	Ref	Ref
Other	0.20(0.01–3.13)	0.23(0.01–5.57)	na	na		
**Wealth quintile**						
Lowest	4.24(2.90–6.21)	2.49(1.46–4.22)	3.83(2.37–6.20)	1.08(0.61–1.92)	4.52(3.56–5.74)	1.97(1.43–2.72)
Second	2.75(1.90–3.99)	1.80(1.13–2.88)	3.01(1.72–5.30)	1.42(0.73–2.76)	3.58(2.84–4.52)	1.86(1.39–2.50)
Middle	2.14(1.49–3.05)	1.61(1.04–2.50)	2.32(1.45–3.72)	1.08(0.61–1.88)	2.60(2.04–3.33)	1.90(1.42–2.55)
Fourth	1.36(0.97–1.90)	1.25(0.80–1.95)	1.71(1.03–2.85)	1.02(0.59–1.74)	1.66(1.29–2.14)	1.30(0.98–1.71)
Highest	Ref	Ref	Ref	Ref	Ref	Ref
**Pseudo R2**		0.205		0.212		0.186

Notes: AOR: adjusted odds ratio; OR: odds ratio

Adjusted for: Residence, education, marital status, religion and wealth quintile

Multicollinearity test was done between wealth and education, the variance inflation factor indicated no collinearity (variance inflation factor < 10)

Ref: Reference category

Div: Divorced

Sep: Separated

### Pooled multivariate regression analysis of socio-demographic determinants of adolescent pregnancy

Table 5 shows factors associated with adolescent pregnancy for the years 2003, 2008/2009, and 2014 combined, with the year of study as one of the covariates. Compared to women with no education, women with incomplete primary education (AOR = 1.66, 95% CI = 1.29–2.14) had a higher likelihood of reporting adolescent pregnancy while those with secondary education (AOR = 0.47, 95% CI = 0.36–0.62) and above had lower odds of adolescent pregnancy. The odds of adolescent pregnancy among married (AOR = 5.38, 95% CI = 4.60–6.30) and divorced (AOR = 6.22, 95% CI = 4.70–8.25) women was higher than those for women who were never married. Religion was also a factor, with Muslim women less likely to report adolescent pregnancy than those with no religion (AOR = 0.62, 95% CI = 0.39–0.98). The likelihood of adolescent pregnancy increased as the household wealth status decreased, with the fourth (AOR = 1.22, 95% CI = 1.01–1.49), middle (AOR = 1.67, 95% CI = 1.38–2.03,), second (AOR = 1.77, 95% CI = 1.45–2.16), and first (AOR = 1.83, 95% CI = 1.47–2.27) household wealth quintiles all having higher odds than the highest household wealth quintile.

**Table 5:** Pooled multivariate regression analysis of socio-demographic determinants of pregnancy during adolescence: (N = 9,141)

	COR(95% CI)	AOR(95% CI)
**Year**		
2003	Ref	Ref
2008/2009	1.01(0.81–1.27)	
2014	0.88(0.76–1.01)	
**Residence**		
Urban	0.51(0.45–0.58)	1.02(0.87–1.21)
Rural	Ref	Ref
**Education**		
No education	Ref	Ref
Primary incomplete	1.50(1.21–1.85)	1.66(1.29–2.14)
Primary complete	0.73(0.60–0.90)	1.03(0.80–1.34)
Secondary +	0.22(0.18–0.27)	0.47(0.36–0.62)
**Marital status**		
Never married	Ref	Ref
Married or living together	7.54(6.48–8.79)	5.38(4.60–6.30)
Divorced/Separated/Widowed	9.02(6.88–11.81)	6.22(4.70–8.25)
**Religion**		
Roman Catholic	0.31(0.20–0.47)	0.81(0.51–1.28)
Protestant/other Christian	0.35(0.24–0.52)	0.81(0.52–1.26)
Muslim	0.40(0.26–0.62)	0.62(0.39–0.98)
No religion	Ref	Ref
Other	0.20(0.01–2.92)	0.32(0.02–6.37)
**Wealth quintile**		
Lowest	4.27(3.53–5.16)	1.83(1.47–2.27)
Second	3.26(2.69–3.95)	1.77(1.45–2.16)
Middle	2.43(2.01–2.94)	1.67(1.38–2.03)
Fourth	1.58(1.30–1.93)	1.22(1.01–1.49)
Highest	Ref	Ref
**Pseudo R2 = 0.1928**		

## Discussion

The analysis presented here investigated adolescent pregnancy trends using retrospective data from 2003 to 2014 DHSs, thereby providing information from across a decade. In addition, we undertook a multivariate regression analysis for the three DHSs to establish the socioeconomic factors associated with pregnancy among adolescents aged 15–19 years old.

There was no reduction in the prevalence of adolescent pregnancy between 2003 and 2008, however, a reduction occurred from 42.2% to 2008/2009 to 38.9% in 2014. The lack of progress in reducing adolescent pregnancy from 2003 to 2008 coincides with a period where the implementation of ASRH programs was not rife in Kenya. An assessment of the 2003 Adolescent Reproductive Health and Development Policy in 2013 revealed that there was limited dissemination of the policy and that it was not implemented equitably to all adolescents in society, especially the hardest to reach and most vulnerable populations [4]. The Performance Monitoring and Accountability (PMA) framework collects nationally representative data on family planning from women of reproductive age in Kenya, among other countries and its methodology is aligned to DHS to allow comparability. PMA 2020 findings shows that adolescent pregnancy in 2018 was 25.6% among women aged 14–18 [8]. These findings indicate a gradual decrease in prevalence from 38.9% to 2014 (our study findings) to 25.6% in 2018 (PMA data) [8]. The findings suggest that sexually active adolescents were using family planning to protect against pregnancy. The use of modern contraceptives among married adolescents rose from 15% to 2003 to 37% in 2014 [17]. The period under analysis also coincides with the launch by the Kenya Ministry of Health and development partners of various policy initiatives aimed at addressing adolescent health needs [4, 23]. Implementation of these policies is likely to have contributed to increased access to ASRH information and services, including the use of contraceptives. An analysis of trends and determinants of adolescent pregnancy and early motherhood in five East African countries using data from the DHS has shown greater improvements in some of the key determinants of adolescent sexual and reproductive health including educational attainment and knowledge and use of contraception [24]. Despite the decrease in adolescent pregnancy, more needs to be done to further reduce this burden, including reducing the unmet need for family planning among sexually active adolescents, which remains high at 23% [17].

The regression analysis indicates that adolescent pregnancy is associated with low education status, lower household wealth quintile, and marriage. Pooled regression analysis also indicated an association between low education status, lower wealth quintile, marriage, and religion (among Catholics and Protestants) and adolescent pregnancy. Systematic reviews have also showed that poverty, and lower educational attainment are consistently associated with adolescent pregnancy [25].

Overall, several factors remained constant over the three years. The odds of reporting adolescent pregnancy remained low among the urban population and high among women who were married or divorced across the three surveys. In addition, education remained a factor among the categories of women with no education and those with secondary education. Education at the primary complete category was a protective factor in 2003 and 2008 but not in 2014. Similar analyses have showed that as adolescents get greater access to education, the opportunities for avoiding early childbearing may improve due to increased knowledge to prevent unintended pregnancies, delayed sexual debut and Marriage [26].

The odds of reporting adolescent pregnancy were present among all wealth quintile categories in 2003 and 2014 and increased from the fourth wealth quintile to the lowest, from 1.71 to 3.73 (2003) and from 1.3 to 1.97 (2014). This relates to findings from an analysis of first births in EastAfrican whose findings revealed very high percentages of adolescent first births among the poorest quintiles for both rural and urban residents [27]. In addition, evidence across the world shows an association between adolescent pregnancy and poverty in both developed and developing countries [28]. While occupation was not investigated in this study, studies have shown that the risk of adolescent pregnancy is higher among adolescent girls in employment, perhaps because female adolescents who are not working may be in school which is a protective factor [29]. We however conducted a multicollinearity test between wealth quintile and occupation and found none.

The association between adolescent pregnancy and low education status is in line with findings from the trend analysis, which showed an increase in women with complete primary education from 41.5% to 2009 to 47.7% in 2014, while the percentage of child marriage decreased from 57.9% to 2003 to 53.9% in 2014. On the other hand, the trend in wealth quintile was not consistent with findings from the regression analysis; the percentage of those in the fourth and highest wealth quintiles, respectively, decreased from 39.6% to 2009 to 33.4% in 2014 and from 27.7% to 2009 to 23.2% in 2014.

Our findings add to the evidence from other studies on the association between education level and adolescent pregnancy [30]. Studies have shown that pregnancy among school-aged girls contributes to school dropout and/or child marriage; an estimated 5–33% of girls aged 15 to 19 years who drop out of school in some countries do so because of early pregnancy or marriage [1]. Increased educational attainment for adolescent girls could bring large poverty reduction benefits in addition to health benefits by avoiding early pregnancies and maternal deaths [31]. The school platform also provides an opportunity to provide ASRH messages and services that would increase the use of ASRH services among sexually active adolescents.

Pregnancy during adolescence was associated with low levels of household wealth, which has a ripple effect on many aspects of affected adolescents who often drop out of school, are forced into child marriage, start childbearing very early and thus end up with many children living in poverty [1]. Increasing the age at first pregnancy is therefore important as it positively impacts the health status of the young mother and baby, but also improves girls’ futures—socially, emotionally, and economically [13].

Our study shows that majority of the 20–24 years old women reported to have been living in rural areas over the three DHS surveys at 69% in 2003, 68.6% in 2008/9 and 51.7% in 2014. It however appears that more women were likely to have been living in urban areas in 2014 at 48.3% as compared to 31% in 2003 and 31.4% in 2008/9. This could be attributed to the devolved system of government enacted in 2010 through a new constitution that created new 47 lower-level county governments. Some of the causes of rural–urban migration includes inequalities, under employment and unemployment in rural areas and perceived income disparities between rural and urban areas among [32]. Devolution in Kenya strengthened urban towns located in rural counties, and where economic investments such as energy and natural resources are largely found [32]. This suggests that towns are attractive for young people in search of economic ventures or in pursuit of higher education opportunities. Various studies have established an association between residence and adolescent pregnancy. An analysis of the 2011 DHS in Uganda established that rural residence was associated with a larger proportion of adolescents (24.8%) either currently pregnant or having born a child, compared to 22% among those in urban areas [33]. A multi-country analysis on prevalence of first adolescent pregnancy and its associated factors in sub-Saharan Africa showed that the odds of having first pregnancy was high among adolescents who lived in rural areas as compared to those in urban areas [34].

Findings from the KDHS 2014 show that although there has been an increase in the modern contraceptive prevalence rate among married adolescents, from 15% to 2003 to 27% in 2008/2009 to 37% in 2014, the unmet need for family planning among adolescents remains high at over 50% [17]. High prevalence of adolescent pregnancy was associated with child marriage, which is likely due to legal, sociocultural, and religious factors. Although 18-year-olds are considered adolescents under the World Health Organization (WHO) definition, the Kenyan constitution states the legal age for adulthood as 18 years, allowing for consent to sex and marriage [35]. This is also the age when the majority of adolescents’ complete secondary education. Consequently, this contributes to a high percentage of adolescents marrying and engaging in sex and becoming pregnant during the adolescence period. Studies have shown that early pregnancy and child marriage, in addition to playing an important part in school dropout rates, are often linked with socioeconomic inequalities and unequal gender norms [36]. Furthermore, child marriage has an economic cost, with countries losing out on the annual income that young women would have earned over their lifetimes if they had not had early pregnancies [1]. Our findings on the factors associated with adolescent pregnancy are consistent with those reported by other studies on adolescent pregnancy in Kenya [2, 14]. Increasing utilization of ASRH information and services among married adolescents is therefore important to delay childbearing and enable birth spacing.

High prevalence of adolescent pregnancy calls for changes at the higher levels of government using a multisectoral approach (especially education), and at the community level where the family unit and community leaders can play a major role. Recent efforts to institutionalize gender equality, uphold the rights of girls, and promote their well-being are likely to bear fruit soon. Article 27 of Kenya’s 2010 Constitution guarantees equality, freedom from discrimination, and equal protection and equal benefit of the law. The 2022/2023 DHS is likely to show evidence of progress made toward achieving United Nations Sustainable Development Goal 5 for gender equality, specifically, elimination of all harmful practices, such as child, early and forced marriage, and female genital mutilations.

Although the enabling policy environment in Kenya provides a platform for ASRH services, the burden of adolescent pregnancy remains high, indicating that more needs to be done. Access to and use of ASRH services by adolescents have the potential to reduce unintended pregnancy and the numerous poor pregnancy outcomes among adolescents, such as preterm births, low-birthweight babies, and complications during childbirth, including death [2, 3, 37]. Explanations for the non-use of contraceptives include limited knowledge of family planning methods, socio-cultural and religious factors prohibiting adolescents from accessing services, unfriendly health service providers, and limited access to family planning products [3, 38]. Since no one intervention can address all these factors, a multifaceted approach must be adopted. There is compelling evidence of effective interventions to improve access to and use of contraceptive information and services by different groups of adolescents in a variety of resource-constrained settings, e.g., incorporating adolescent-focused clinics in the existing health care delivery systems [39]. Studies from various parts of the world have shown that adolescent focused SRH programs are effective in reducing adolescent pregnancy and addressing the unmet need for family planning [1]. WHO also recommends that countries collect, analyse, and use accurate and up-to-date data on adolescent sexuality and health outcomes to inform the development of laws, policies, and strategies that are responsive to the needs of different groups of adolescents, based on their social and economic status [1].

### Study limitations

Although the findings show a reduction in adolescent pregnancy from 2008 to 2014, DHSs are cross-sectional in design and therefore have an inherent inability to establish a causal relationship since data on exposure and outcome variables are collected at the same time. While there are other documented determinants of adolescent pregnancy, our study focused on selected socio demographic characteristics. This study has most likely underestimated the prevalence of adolescent pregnancy, since data collected is limited to current pregnancies and previous live births, and do not capture adolescent pregnancies that end in miscarriage, abortion, or stillbirth. Furthermore, the correlates we used in the secondary data analysis are not likely to be comprehensive in analysing all factors relating to the health care systems or the attitude of adolescents toward SRH. In addition, although WHO defines adolescent pregnancy as any pregnancy before age 20, in the trend analysis and logistic regression, we used retrospective data from women who were 20–24 years old. We note that prior studies recommend caution in interpreting adolescent reproductive transitions based on retrospective survey data, due to possible recall and social desirability bias, and possible errors in imputation. In addition, the characteristics of interest included in the analysis may have been different at the actual time of pregnancy (when participants were 15–19 years) compared to the timing of the analysis (when participants are 20–24 years). Finally, this study purposely focused on adolescent’s pregnancy at ages 15–19 years, leaving out girls aged 10–14 years. Further research on the sociocultural factors that fuel adolescent pregnancy is recommended. Analysis of adolescent pregnancy trends by geography is recommended for future studies.

### Implication for policy and program

Findings from this study provide an opportunity to track past progress and therefore, inform Current and future programming. Since education status, marital status, and wealth quintile were associated with adolescent pregnancy, interventions to keep girls in school, alleviation of household poverty and elimination of child marriage need to be intensified. This calls for a multi-sectoral and multi-pronged approaches to addressing SRH issues among adolescents. Continuous data analyses will be key to monitor progress towards addressing the burden of adolescent pregnancy. To further reduce adolescent childbearing, provision of accurate ASRH information and services that can contribute to reducing the burden.

## Conclusion

This study shows a reduction in adolescent pregnancy rates over a decade. Although marginal, the declining trend in adolescent pregnancy rates indicates a need for concerted efforts to further reduce adolescent pregnancy. Understanding the socio-cultural and demographic factors that influence adolescent pregnancy is important in designing appropriate interventions to address these challenges. Strengthening reproductive health services and programs targeting adolescents throughout the continuum of care and using a multisectoral approach will likely contribute to better health outcomes for adolescents.

## Electronic supplementary material

Below is the link to the electronic supplementary material.

Supplementary Material 1

Supplementary Material 2

## Data Availability

The datasets used and/or analysed during the current study are available from the corresponding author on reasonable request. The KDHS data sets used in this study are also available at https://dhsprogram.com/data/available-datasets.cfm.

## Declarations

### Accordance

This study was based on secondary analysis of publicly available demographic health survey data hence did not touch on human subjects. Procedures and questionnaires for standard DHS surveys have been reviewed and approved by ICF Institutional Review Board (IRB). For this secondary analysis, the study was guided by a protocol approved by the Kenya Medical Research Institute research ethics review committee number 597.

### Disclaimer

The views and opinions expressed in this paper are those of the author and not necessarily the views and opinions of the US Agency for International Development (USAID).

## Abbreviations

AOR: Adjusted Odds Ratio.
ASRH: Adolescent Sexual Reproductive Health.
CI: Confidence Interval.
DHS: Demographic Health Survey.
OR: Odds Ratio.
VIF: Variation Inflation Factor.
WHO: World Health Organization.
