# Supplementary material for: Trends and determinants of adolescent pregnancy: Results from Kenya demographic health surveys 2003–2014
Source: BMC Womens Health. 2022 Oct 10;22:416. doi: 10.1186/s12905-022-01986-6 (PMC9552415; doi:10.1186/s12905-022-01986-6)
Supplement: Supplementary file 1 — Supplementary Material 1 [file 12905_2022_1986_MOESM1_ESM.docx]

| **Supplementary file 1: Percentage of women aged 20–24 years who experienced pregnancy during adolescence (ages 10-14years)** | | | | | | | |
| --- | --- | --- | --- | --- | --- | --- | --- |
|  | **2003 (N=1,691)** |  | **2008/2009 (N=1,715)** |  | **2014 (N=5,735)** |  |  |
|  | **N** | **% (95% CI)** | **n** | **% (95% CI)** | **n** | **% (95% CI)** | **Trend 2003-2014** |
| Prevalence of pregnancy (10-14) | 1691 | 3.4(2.6-4.4) | 1714 | 4.5(3.6-5.6) | 5,735 | 4.1(2.8-4.1) | 1.37(0.94-2.00) |
| **Residence** |  |  |  |  |  |  |  |
| Urban | 524 | 3.1(1.9-4.9) | 539 | 2.2(1.2-3.8) | 2769 | 3.4(4.1-5.6) |  |
| Rural | 1168 | 3.6(2.6-4.8) | 1175 | 5.6(4.4-7.1) | 2966 | 4.8(4.1-5.6) |  |
| **Education** |  |  |  |  |  |  |  |
| No education | 124 | 11.7(7.1-18.6) | 124 | 14.4(9.2-21.8) | 301 | 13.1(9.7-17.4) |  |
| Primary incomplete | 469 | 4.9(3.2-7.2) | 426 | 9.0(6.6-12.1) | 1015 | 8.9(7.3-10.8) |  |
| Primary complete | 521 | 3.2(1.9-5.1) | 504 | 3.4(2.1-5.4) | 1353 | 4.1(3.2-5.3) |  |
| Secondary+ | 578 | 0.7(0.3-1.8) | 661 | 0.6(0.2-1.6) | 3066 | 1.7(1.3-2.2) |  |
| **Household wealth quintile** | |  |  |  |  |  |  |
| Lowest | 221 | 5.0(2.8-8.8) | 253 | 13.5(9.8-18.3) | 809 | 8.0(6.3-10.1) |  |
| Second | 264 | 5.3(3.2-8.8) | 267 | 1.9(0.8-4.4) | 973 | 6.2(4.9-7.9) |  |
| Middle | 294 | 3.7(2.0-6.6) | 296 | 6.5(4.2-9.9) | 998 | 3.0(2.1-4.3) |  |
| Fourth | 363 | 3.0(1.7-5.4) | 353 | 2.9(1.6-5.2) | 1290 | 3.7(2.8-4.9) |  |
| Highest | 550 | 1.9(1.1-3.5) | 546 | 1.6(0.8-3.1) | 1665 | 2.0(1.4-2.8) |  |
| **Religion** |  |  |  |  |  |  |  |
| Catholic | 411 | 4.8(3.1-7.3) | 359 | 2.9(1.5-5.2) | 1186 | 3.7(2.8-5.0) |  |
| Protestant/Other Christian | 1125 | 2.8(2.0-4.0) | 1190 | 4.0(3.0-5.3) | 4,091 | 4.3(3.7-4.9) |  |
| Muslim | 115 | 4.0(1.6-9.6) | 122 | 8.6(4.7-15.0) | 351 | 3.6(2.1-6.1) |  |
| No religion | 33 | 5.2(1.1-20.8) | 38 | 23.7(12.7-39.8) | 89 | 4.8(1.9-1.2) |  |
| Other | 4 | NA | 3 | NA | 10 | 4.4(0.2-52.2) |  |
| **Marital status** |  |  |  |  |  |  |  |
| Never married | 1612 | 1.3(0.6-2.5) | 651 | 0.8(0.3-1.8) | 2225 | 1.2(0.8-1.8) |  |
| Married/living with partner | 965 | 4.1(3.0-5.5) | 958 | 6.5(5.1-8.2) | 3,133 | 5.9(5.1-6.7) |  |
| Widowed/divorced/separated | 115 | 9.3(5.2-16.2) | 106 | 9.7(5.3-17.0) | 377 | 6.7(4.6-9.7) |  |
