# Supplementary material for: Trends and determinants of adolescent pregnancy: Results from Kenya demographic health surveys 2003–2014
Source: BMC Womens Health. 2022 Oct 10;22:416. doi: 10.1186/s12905-022-01986-6 (PMC9552415; doi:10.1186/s12905-022-01986-6)
Supplement: Supplementary file 2 — Supplementary Material 2 [file 12905_2022_1986_MOESM2_ESM.docx]

| **Supplementary 2: Pooled multivariate regression analysis of sociodemographic determinants of pregnancy during adolescence: (10-14)** | | |
| --- | --- | --- |
|  | **COR(95% CI)** | **AOR (95% CI)** |
| **Year** |  |  |
| **2003** | Ref | Ref |
| 2008/2009 | 1.34(0.87-2.06) |  |
| 2014 | 1.22(0.83-1.78) |  |
| **Residence** |  |  |
| Urban | 0.67(0.48-0.92) | 1.81(1.27-2.60) |
| Rural | Ref | Ref |
| **Education** |  |  |
| No education | Ref | Ref |
| Primary incomplete | 0.57(0.42-0.79) | 0.50(0.34-0.73) |
| Primary complete | 0.26(0.18-0.38) | 0.25(0.16-0.39) |
| Secondary + | 0.09(0.06-0.14) | 0.12(0.07-0.20) |
| Marital status |  |  |
| **Never married** | Ref | Ref |
| Married or living together | 5.15(3.31-8.03) | 2.88(1.79-4.61) |
| Divorced/Separated/Widowed | 7.25(4.10-12.80) | 3.91(2.18-6.99) |
| **Religion** |  |  |
| Roman Catholic | 0.38(0.20-0.72) | 1.24(0.65-2.36) |
| Protestant/other Christian | 0.40(0.22-0.71) | 1.18(0.66-2.14) |
| Muslim | 0.48(0.25-0.91) | 0.52(0.27-1.01) |
| No religion | Ref | Ref |
| Other | 0.13(0.01-1.87) | NA |
| **Wealth quintile** |  |  |
| Lowest | 4.85(3.10-7.59) | 2.56(1.43-4.59) |
| Second | 2.88(1.82-4.55) | 2.21(1.27-3.84) |
| Middle | 2.03(1.22-3.37) | 1.82(1.04-3.21) |
| Fourth | 1.83(1.03-3.26) | 1.71(0.90-3.26) |
| Highest | Ref | Ref |
| Pseudo R2=0.1050 |  |  |
